# Identification and verification of the optimal feature genes of ferroptosis in thyroid-associated orbitopathy

**DOI:** 10.3389/fimmu.2024.1422497

**Published:** 2024-12-13

**Authors:** Xuemei Li, Chao Xiong, Siyi Wang, Zhangjun Ren, Qi Jin, Jinhai Yu, Yunxiu Chen, Puying Gan, Qihua Xu, Yaohua Wang, Hongfei Liao

**Affiliations:** ^1^ School of Optometry, Jiangxi Medical College, Nanchang University, Nanchang, Jiangxi, China; ^2^ Department of Ophthalmology, The Affiliated Eye Hospital, Jiangxi Medical College, Nanchang University, Nanchang, Jiangxi, China; ^3^ Jiangxi Clinical Research Center for Ophthalmic Disease, Nanchang, Jiangxi, China; ^4^ Jiangxi Research Institute of Ophthalmology and Visual Science, Nanchang, Jiangxi, China; ^5^ Jiangxi Provincial Key Laboratory for Ophthalmology, Nanchang, Jiangxi, China

**Keywords:** orbitopathy, ferroptosis-related gene, immune cell infiltration, GEO, WGCNA

## Abstract

**Background:**

Thyroid-associated orbitopathy (TAO) is an autoimmune inflammatory disorder of the orbital adipose tissue, primarily causing oxidative stress injury and tissue remodeling in the orbital connective tissue. Ferroptosis is a form of programmed cell death driven by the accumulation of reactive oxygen species (ROS), iron metabolism disorder, and lipid peroxidation. This study aims to identify and validate the optimal feature genes (OFGs) of ferroptosis with diagnostic and therapeutic potential in TAO orbital adipose tissue through bioinformatics analysis and to assess their correlation with disease-related immune cell infiltration.

**Methods:**

Search of the Gene Expression Omnibus database for TAO-related gene datasets led to the selection of GSE58331 for differential gene expression analysis. WGCNA was employed to identify key disease modules and hub genes. The intersection of DEGs, hub genes and ferroptosis-related gene yielded key genes of ferroptosis. Machine learning algorithms identified OFGs of ferroptosis. Meanwhile, by comparing the expression of FRGs in the orbital adipose tissue and the orbital fibroblasts (OFs) of healthy controls and TAO patients, as well as co-culturing macrophages and OFs *in vitro*, the influence of macrophages on FRGs in OFs was explored. CIBERSORT analyzed immune cell infiltration to determine proportions of immune cell types in each sample, and Spearman correlation analysis explored relationships between OFGs and infiltrating immune cells. Finally, GSEA determined the function of each key biomarker based on the median expression of OFGs.

**Results:**

Three TAO FRGs (ACO1, MMD, and HCAR1) were screened in the dataset. The ROC results of ACO1 showed that the AUC value was greater than 0.8 in all the datasets, which was the strongest for disease specificity and diagnostic ability. Validation results showed that, in addition to MMD, the expression of ACO1 and HCAR1 in orbital adipose tissue of TAO patients was significantly down-regulated, while M2-type macrophages might be involved in regulating the expression of ACO1 in orbital adipose-derived OFs. CIBERSORT immune cell infiltration analysis showed that in orbital adipose tissue of TAO patients, memory B-lymphocytes, T regulatory cells, NK-cells, M0-type macrophages, M1-type macrophages, resting dendritic cells, activated mast cells, and neutrophils infiltration levels were significantly elevated.

**Conclusion:**

Through bioinformatics analysis, this study identified and validated two OFGs of ferroptosis with diagnostic and therapeutic potential in TAO orbital adipose tissue, suggesting that the downregulation of ACO1 and HCAR1 may be potential molecular targets in the pathogenesis of TAO.

## 1 Introduction

TAO is an autoimmune orbital disease associated with the thyroid gland that occurs in the eye and retrobulbar tissues and poses a serious threat to visual function, affects ocular appearance, and has very low quality of life scores (1, 2). TAO is an orbital autoimmune inflammatory disease that can lead to a variety of pathologic changes in orbital connective tissues (including the transverse muscle, smooth muscle, adipose tissue, lacrimal glands, and fascial system), such as inflammatory infiltration, retrobulbar fat hyperplasia, and thickening of extraocular muscles (3, 4). Clinically, the typical course of TAO is divided into an initial phase of inflammatory activity and a subsequent phase of inflammatory quiescence (1). Various medications and radiation treatments for TAO are currently available with good results for some patients, but a high percentage of patients still end up needing multiple reconstructive eye surgeries due to poor treatment outcomes.

Previous studies have shown that oxidative stress and orbital lipoatrophy are two key pathophysiological changes in patients with TAO, found that target tissues and target cells are generally in a state of oxidative stress, and proposed that oxidative stress *in vivo* (i.e., a state of imbalance in the oxidative/antioxidant system) plays a crucial role in the course and progression of TAO (3, 5). In the active phase of TAO, the most prominent pathologic change is the immunoinflammatory reaction of the orbital adipose tissue, and its inflammatory changes are manifested by the infiltration of various immune cells in the orbital adipose tissue (6). Studies on the pathogenesis of resting TAO with proptosis and diplopia have found that connective tissue remodeling is the main pathological change, including hyperplasia of orbital adipose tissue and fibrosis of extraocular muscles (5, 7–9). However, the specific pathogenesis of TAO remains unclear. Ferroptosis is a newly identified form of programmed cell death in recent years, characterized by the accumulation of ROS inside cells and lipid peroxidation induced by the iron-dependent high expression of unsaturated fatty acids on the cell membrane, leading to cell death (10–12). Current research suggests that ferroptosis is involved in the occurrence and development of various diseases, including cancer, inflammatory diseases, autoimmune diseases, cardiovascular diseases, metabolic diseases, and neurodegenerative diseases (13). Although there is considerable research on the role of ferroptosis in the pathogenesis of diseases and drug development, its relationship with TAO is still in the early stages.

In the human genome, only 2%-3% of transcriptionally active RNAs have the capability to encode proteins (14, 15). Recent studies utilizing high-throughput sequencing and bioinformatics analysis of orbital adipose tissue have identified differential messenger RNA (mRNA) expression associated with the pathogenesis of TAO (16, 17). However, there is limited research on the specific regulatory mechanisms by which differentially expressed genes(DEGs) in TAO orbital adipose tissue contribute to the disease mechanism. In recent years, the role of ferroptosis in the pathogenesis of TAO has garnered increasing attention. This study aims to identify the OFGs of ferroptosis in TAO orbital adipose tissue through bioinformatics analysis, WGCNA, and three machine learning methods. Furthermore, it explores the expression of FRGs in the orbital adipose tissue of TAO patients and their significance as diagnostic markers and potential therapeutic targets for the disease.

## 2 Materials and methods

**Figure 1:**
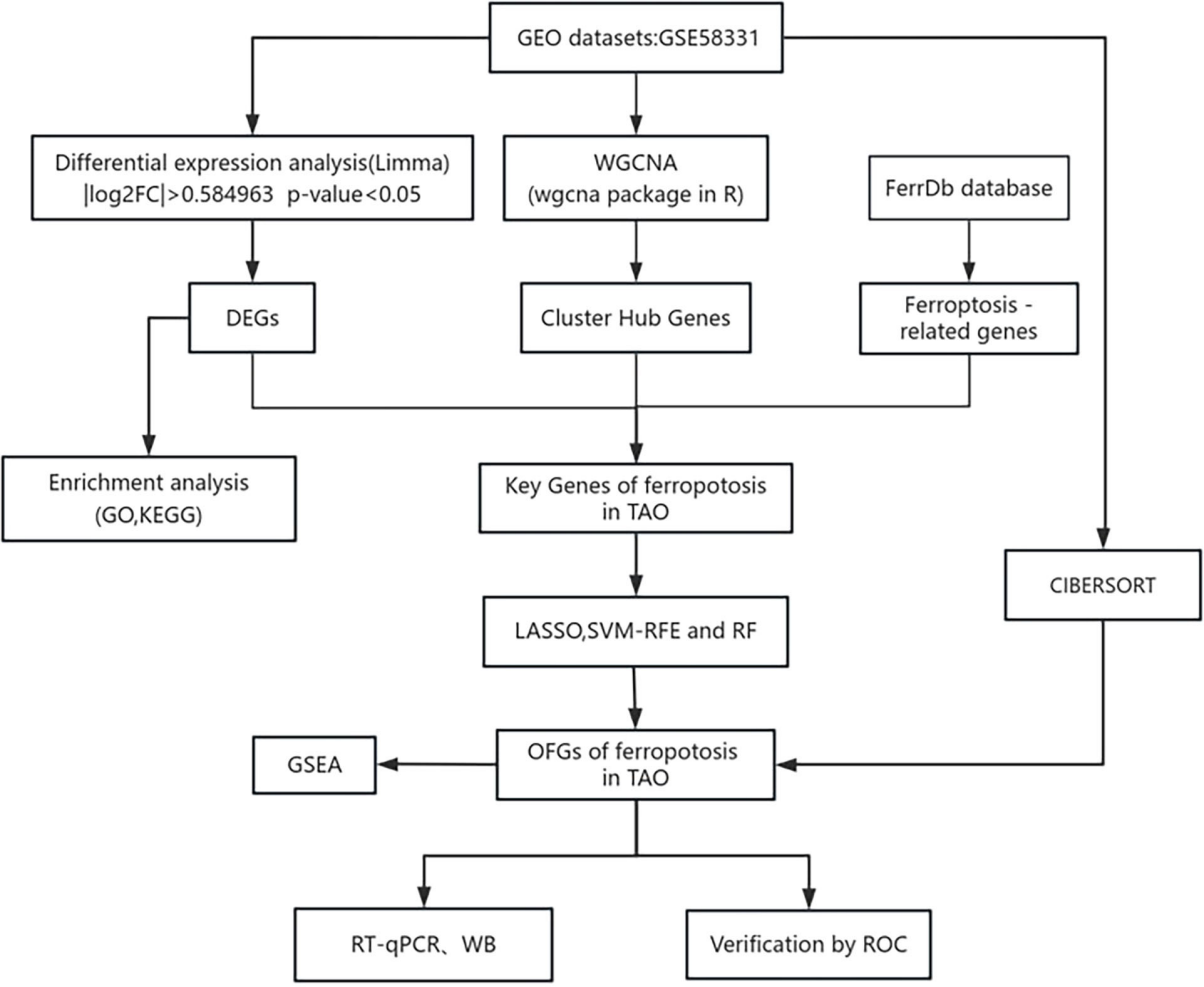
The flow chart of this study.

In a search of the GEO database using the keyword “Graves ophthalmopathy,” we acquired the dataset GSE58331 for subsequent analyses (15). This dataset comprises 175 samples of anterior orbital or lacrimal gland tissues from healthy individuals as well as patients with inflammatory diseases such as NSOI, sarcoidosis, GPA, and TAO. In our study, preorbital adipose tissue samples from 27 TAO patients and samples from 22 healthy individuals were selected, and the lower figure shows the flow chart of this study (**Figure 1**).

### 2.1 Screening for DEGs

Using R (version 4.2.0), the expression matrices of 49 samples (normal group and TAO group) were subjected to data correction and quantile normalization. Subsequently, genes with P < 0.05 and |log2FC| >0.584963, corresponding to a 1.5-fold change, were identified as DEGs. Heatmaps and volcano plots were then constructed to visualize these results.

### 2.2 GO and KEGG enrichment analysis

DEGs were analyzed for enrichment using the Gene Ontology (GO) and Kyoto Encyclopedia of Genes and Genomes (KEGG) pathways (18). Metascape (http://metascape.org/) is a powerful tool for gene function annotation and analysis. We separately imported the up-regulated and down-regulated differentially expressed genes into Metascape for online analysis to obtain the corresponding GO and KEGG enrichment analysis results.

### 2.3 WGCNA analysis

WGCNA was employed to identify highly co-expressed gene sets and potential biomarkers (19). The co-expression network for GSE58331 was constructed using the “WGCNA” package in R, with the minimum number of genes per module set at 30. Key modules were identified using correlation analysis, and genes within these key modules were designated as hub genes.

### 2.4 TAO and ferroptosis -related genes

Genes associated with ferroptosis, categorized as “Driver,” “Suppressor,” and “Marker,” were retrieved from the FerrDb database (http://zhounan.org/ferrdb/) (20). The key genes for ferroptosis of TAO were obtained by intersection of DEGs, hub genes and ferroptosis related genes and visualized by Venn diagram.

### 2.5 The OFGs of ferroptosis in TAO

Three machine learning methods were employed to further screen the TAO key genes associated with ferroptosis. To determine the optimal penalty value with the minimal binomial bias, we conducted LASSO logistic regression analysis using the “glmnet” package in R, which involves the least absolute shrinkage and selection operator. Support Vector Machine Recursive Feature Elimination (SVM-RFE) was performed using R packages “e1071,” “kernlab,” and “caret,” focusing on minimizing cross-validation error. The Random Forest approach, utilizing the “randomForest” package in R, was developed to determine the level of minimal error. The intersection of results from these three machine learning algorithms identified OFGs for ferroptosis.

### 2.6 Verification of the OFGs of ferroptosis in TAO by ROC curve

Using the “pROC” tool, a receiver operating characteristic (ROC) curve was created to evaluate the diagnostic significance of the top candidate genes associated with ferroptosis in TAO. The area under the ROC curve (AUC) indicates the diagnostic performance.

### 2.7 Immune infiltration analysis

The CIBERSORT algorithm was used for immune infiltration analysis, which involves analyzing standardized gene expression data obtained from the CIBERSORT algorithm to determine the proportions of 22 immune cell types in each sample. Spearman correlation analysis in R was then employed to explore the potential relationship between the top candidate genes associated with ferroptosis in TAO and infiltrating immune cells.

### 2.8 GSEA analysis

Based on the median expression of the top candidate genes associated with ferroptosis in TAO, samples were divided into high and low expression groups. The processed fold change values were then sorted in descending order to represent the trend of gene expression changes between the two groups, aiming to more accurately determine the functional significance of each key biomarker.

### 2.9 Orbital adipose tissue verified FRGS

#### 2.9.1 Verification of the OFGs of ferroptosis in TAO by RT-qPCR

Three TAO patients with orbital decompression surgery who were admitted to the Affiliated Eye Hospital of Nanchang University and diagnosed with thyroid-related eye disease according to the 22-year old Chinese guidelines for the diagnosis of thyroid-related eye disease were selected. They were between 18 and 65 years old and had a stable eye condition and thyroid function for more than 6 months. Additionally, 3 patients who underwent double eyelid surgery and eyebag plastic surgery in our hospital from October 2023 to December 2023 were selected. This study followed the guidelines of the Helsinki Ethics Committee and was approved by the Affiliated Eye Hospital of Nanchang University (file No. YLS20231020). Total RNA was extracted from orbital adipose tissue using the AG RNAex Pro RNA Extraction Kit (AG21101, ACCURATE BIOTECHNOLOGY (HUNAN) CO., LTD., Changsha, China), and purified with the Steady Pure RNA Extraction Kit (AG21024, ACCURATE BIOTECHNOLOGY (HUNAN) CO., LTD., Changsha, China). The purified RNA was reverse-transcribed into cDNA using the Evo M-MLV RT PreMix Kit (AG11728, ACCURATE BIOTECHNOLOGY (HUNAN) CO., LTD., Changsha, China) for qPCR analysis. Primer sequences for three FRGs and β-actin, used as a housekeeping gene, are listed in **Table 1**. Amplification was performed using the SYBR Green Pro Taq HS qPCR Kit (AG11701, ACCURATE BIOTECHNOLOGY (HUNAN) CO., LTD., Changsha, China), with fluorescence signals collected and analyzed during the exponential amplification phase. All results were analyzed based on three independent experiments, each with triplicate assays.

**Table 1:** Primer name and sequence.

Primer name	Primer Sequences
ACO1-Forward primer	5′-CAAGCAGGCACCACAGACTA-3′
ACO1-Reverse primer	5′-AGGACGGCTTTGATTCCCAG-3′
MMD-Forward primer	5′-AATGGGACTCTGTGCCCTCT-3′
MMD-Reverse primer	5′-GGGGTCCAAGTTCACGAAGA-3′
HCAR1-Forward primer	5′-TCGTGTTCCTTACGGTGGTG-3′
HCAR1-Reverse primer	5′-TTGCACGCAGAGATGGTTCT-3′
β-actin-Forward primer	5′-TGGCACCCAGCACAATGAA-3′
β-actin -Reverse primer	5′-CTAAGTCATAGTCCGCCTAGAAGCA-3′

#### 2.9.2 The expression of FRGS in orbital adipose tissue was verified by WB

Orbital adipose tissue was obtained from TAO patients undergoing orbital decompression surgery (n=3) and healthy subjects undergoing ocular plastic surgery (n=3) in the Affiliated Eye Hospital of Nanchang University. Orbital adipose tissue was lysed with RIPA buffer (Solarbio, China) to extract protein, which was transferred to PVDF membrane by SDS-PAGE gel electrophoresis. PVDF membrane was immersed in 5% skim milk powder and blocked for 1.5h. The PVDF membrane was then incubated overnight in a shaking bed at 4°C in ACO1 antibody (PA5-41753, Thermofisher), HCAR1 antibody (ER62150, HuaBio), and MMD antibody (A06022, Boster) prepared according to the corresponding dilution ratio. The protein bands were then developed by WesternBright ECL (K-12045-D10, Advansta, USA) after incubation at room temperature for 1 hour. Automatic chemiluminescence image analysis system (Tanon-5200Multi, China) was used for detection, and band quantification was performed by ImageJ software.

### 2.10 Verification of cell experiments

#### 2.10.1 Culture and identification of orbital fat derived OFs

The adipose tissue obtained during the operation is placed in a sterilized 15 mL centrifuge tube and transported to the laboratory as soon as possible. The tissue will be cut with scissors into fat pieces scattered about the size of 1 mm³ in petri dishes to join complete medium (DMEM (Vivacell Biosciences C3110-1500, China) + 20% FBS (10099-141 - c, Gibco, Australia) + 1% Penicillin-Streptomycin Liquid (P1400, Solarbio, China); After about 3-7 days, the cells around the tissue block can be seen to swim out, and the cells around the small tissue block can be removed when the cells around the small tissue block swim out gradually. Approximately 80%-90% of the cells had grown by 5-7 days after the tissue block was removed, and cells from generations 3-7 were used for the first passage. The cells were implanted into the 24-well plate, and when the cells grew to 70%-80%, they were fixed with ice methanol, and blocked at room temperature with 3% BSA (A8020, Solarbio, China) for 1 hour. The samples were incubated with α-SMA antibody (14395-1-AP, Proteintech, China), Fibronectin antibody (15163-1-AP, Proteintech, China) and Vimentin antibody (60330-1-Ig, Proteintech, China), and Cytokeratin-19 antibody (10712-1-AP, Proteintech, China) were incubated overnight in a wet box at 4°C, then incubated with fluorescein II antibody at room temperature for 1 hour and then with DAPI at room temperature for 10 minutes. Finally, the images were taken by confocal microscope after the anti-fluorescence quencher was sealed.

#### 2.10.2 Orbital adipogenic OFs verified FRGS

The fifth-generation orbital adipogenic fibroblasts were lysed with RIPA buffer (Solarbio, China) and then transferred to PVDF membrane by SDS-PAGE gel electrophoresis. The PVDF membrane was immersed in 5% skim milk powder and blocked for 1.5h. The PVDF membrane was then incubated overnight on a shaking incubator at 4°C in ACO1 antibody (PA5-41753, Thermofisher), HCAR1 antibody (ER62150, HuaBio) and MMD antibody (A06022, Boster) prepared according to the corresponding dilution ratio. The protein bands were then developed by WesternBrightECL (Advansta, USA) after incubation at room temperature for 1 hour. Automatic Chemiluminescence Image Analysis System (Tanon-5200 Multi, China) was used for detection, and band quantification was performed by ImageJ software.

#### 2.10.3 Identification of induced M0 and M2 macrophages *in vitro*

The THP-1 cells in this study were cultured using DMEM complete medium (containing 10% FBS and 1% Penicillin-Streptomycin Liquid). When the cell density reached 80%, the cells were collected in a six-well plate at a density of about 3.5×10^5 cells/mL. At the same time, 100 ng/mL PMA was added, and the cells were induced to differentiate into macrophages after 48 h PMA treatment in a cell incubator. RNA was collected and extracted from the cells. According to the above method, macrophages formed by PMA-induced differentiation were washed three times with PBS, and DMEM containing IL-4 (20 ng/mL) and IL-13 (20 ng/mL) was added to complete media for 72 h, and RNA was collected from the cells. The macrophage surface molecule CD11b was verified using RT-PCR. M2 macrophage surface molecule: CD163. See **Table 2** for details.

**Table 2:** Primer name and sequence.

Primer name	Primer Sequences
CD11b-Forward primer	5’-CAGGTTCTGGCTCCTTCCA-3’
CD11b-Reverse primer	5’-TCCAACCACCACCCTGGAT-3’
CD163-Forward primer	5’-TTTGTCAACTTGAGTCCCTTCAC-3’
CD163-Reverse primer	5’-TCCCGCTACACTTGTTTTCAC-3’
β-actin-Forward primer	5’-TGGCACCCAGCACAATGAA-3’
β-actin -Reverse primer	5’-CTAAGTCATAGTCCGCCTAGAAGCA-3’

#### 2.10.4 Macrophages were co-cultured with orbital fat derived OFs

After the differentiation of THP-1 cells into M0 macrophages was induced according to the above method, the cells were washed 3 times with PBS, fresh DMEM was added to the complete medium, and the supernatant was collected after overnight incubation in the cell culture box. After M0 macrophages were induced to polarize into M2 macrophages, the cells were washed 3 times with PBS, fresh DMEM was added to the complete culture medium, and the supernatant was collected overnight. The fifth-generation orbital OFs were seeded into the six-well plate, and when the cell size reached 80%-90%, the cells were cultured with normal DMEM complete medium, M0 supernatant, and M2 supernatant for 72 hours. The cells were then collected, and RNA and protein were extracted, respectively, for follow-up verification of the expression of ACO1, MMD, and HCAR1.

## 3 Result

### 3.1 Identification of DEGs

**Figure 2:**
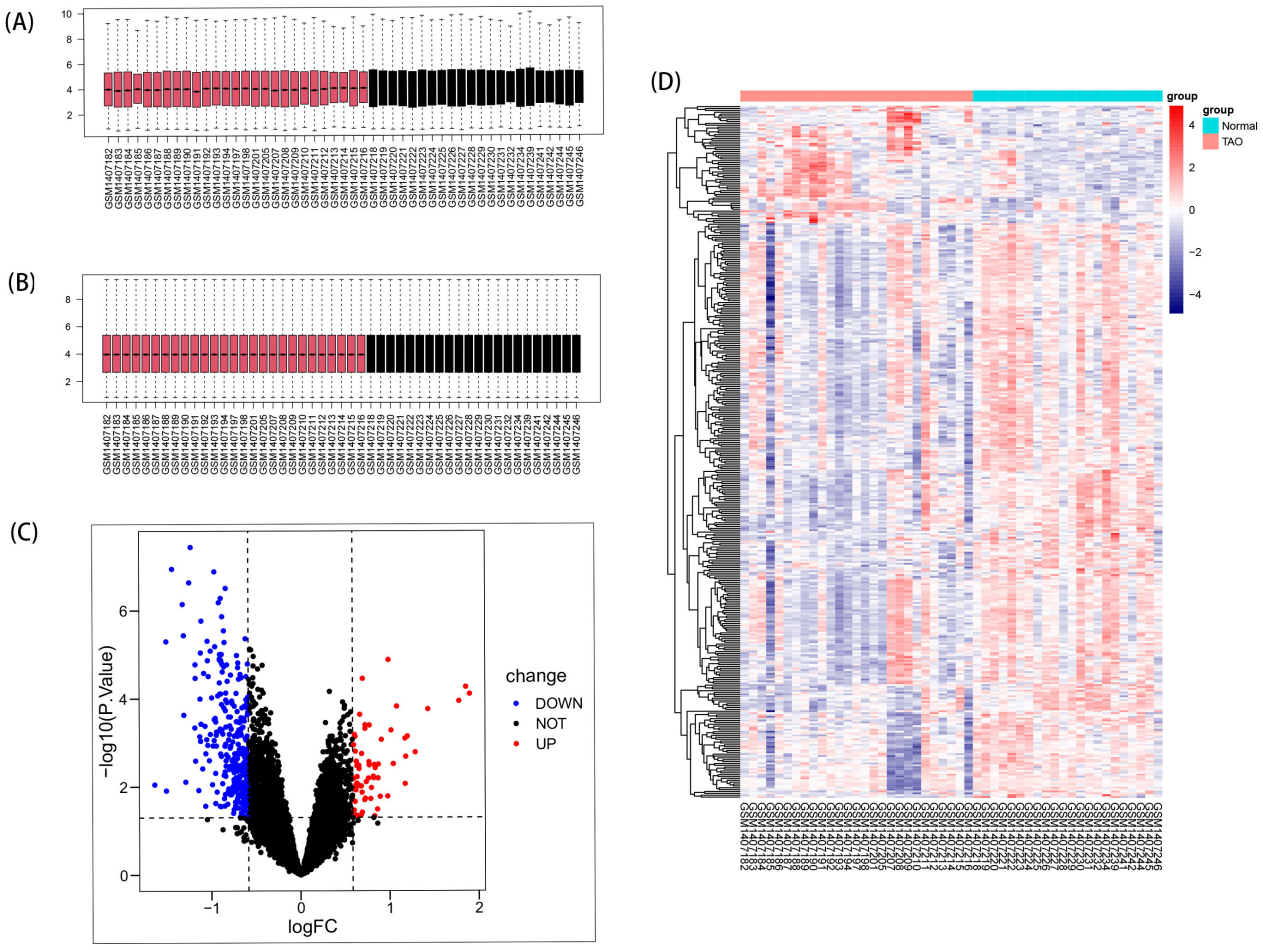
DEGs in anterior orbital adipose tissue between the TAO and Normal individuals. **(A)** Box plots of included samples before removing batch effects. **(B)** Box plots of included samples after removing batch effects. **(C)** Volcano plot of DEGs. Data points in red are up-regulated genes, and in blue are down-regulated genes. **(D)** The heatmap shows the clustering of DEGs in the normal and TAO groups, with red squares representing up-regulation, blue squares representing down-regulation, and the darker color represents the higher fold of differential expression.

Remove data correction batch effect (**Figures 2A, B**), and then based on the inclusion criteria of DEGs with a significance level of P < 0.05 and |log2FC| >0.584963, a total of 366 DEGs were identified from dataset GSE58331, comprising 69 upregulated and 297 downregulated genes. These findings were visually presented using volcano plots (**Figure 2C**) and heatmaps (**Figure 2D**).

### 3.2 Functional enrichment analyses

**Figure 3:**
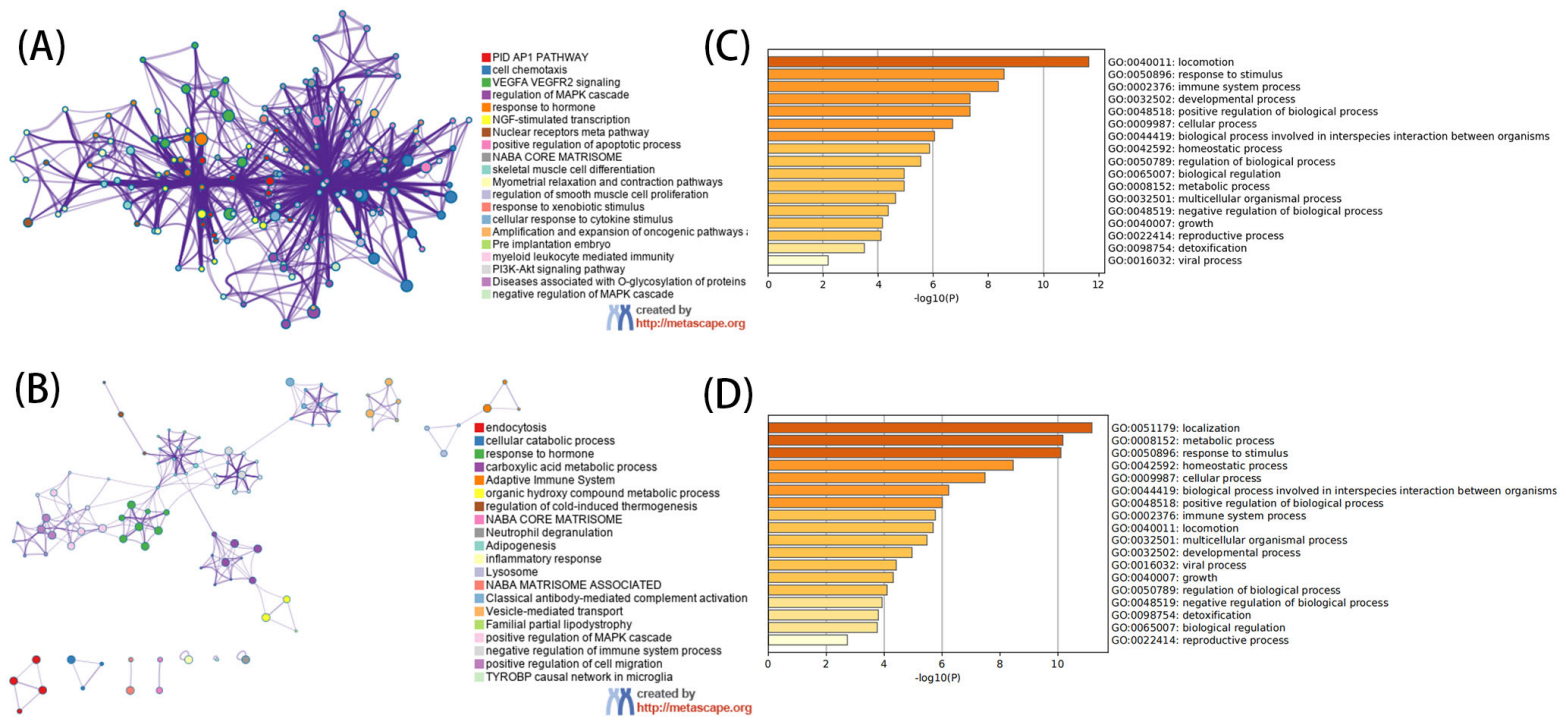
Functional analysis of DEGs. **(A)** For GO enrichment analysis of up-regulated DEGs. **(B)** For GO enrichment analysis of down-regulated DEGs. **(C)** For KEGG enrichment analysis of up-regulated DEGs. **(D)** For KEGG enrichment analysis of down-regulated DEGs. **(A, C)** use network graphs to display the correlation between enriched pathways, with pathways having a correlation greater than 0.3 connected by lines, and different colors representing different enriched pathways. The length of the bar corresponds to the scale after transformation by log10(P) into the enriched P value, with a longer bar and darker color indicating a more significant enrichment of that function.

The GO analysis, encompassing biological processes (BP), cellular components (CC), and molecular functions (MF), revealed that DEGs primarily participate in BP such as cell chemotaxis, responses to peptide hormone, and responses to oxygen levels. CC includes collagen-containing extracellular matrix, secretory granule lumen, cytoplasmic vesicle lumen, and vesicle lumen. MF involves extracellular matrix structural constituent, growth factor binding, glycosaminoglycan binding, heparin binding, and cytokine binding (**Figures 3A, B**). KEGG analysis showed that the up-regulated genes were mainly involved in locomotion and were enriched. Further analysis of down-regulated genes mainly focused on localization, metabolic processes, response to stimulus, homeostatic processes, and other pathways (**Figures 3C, D**).

### 3.3 WGCNA analysis results

**Figure 4:**
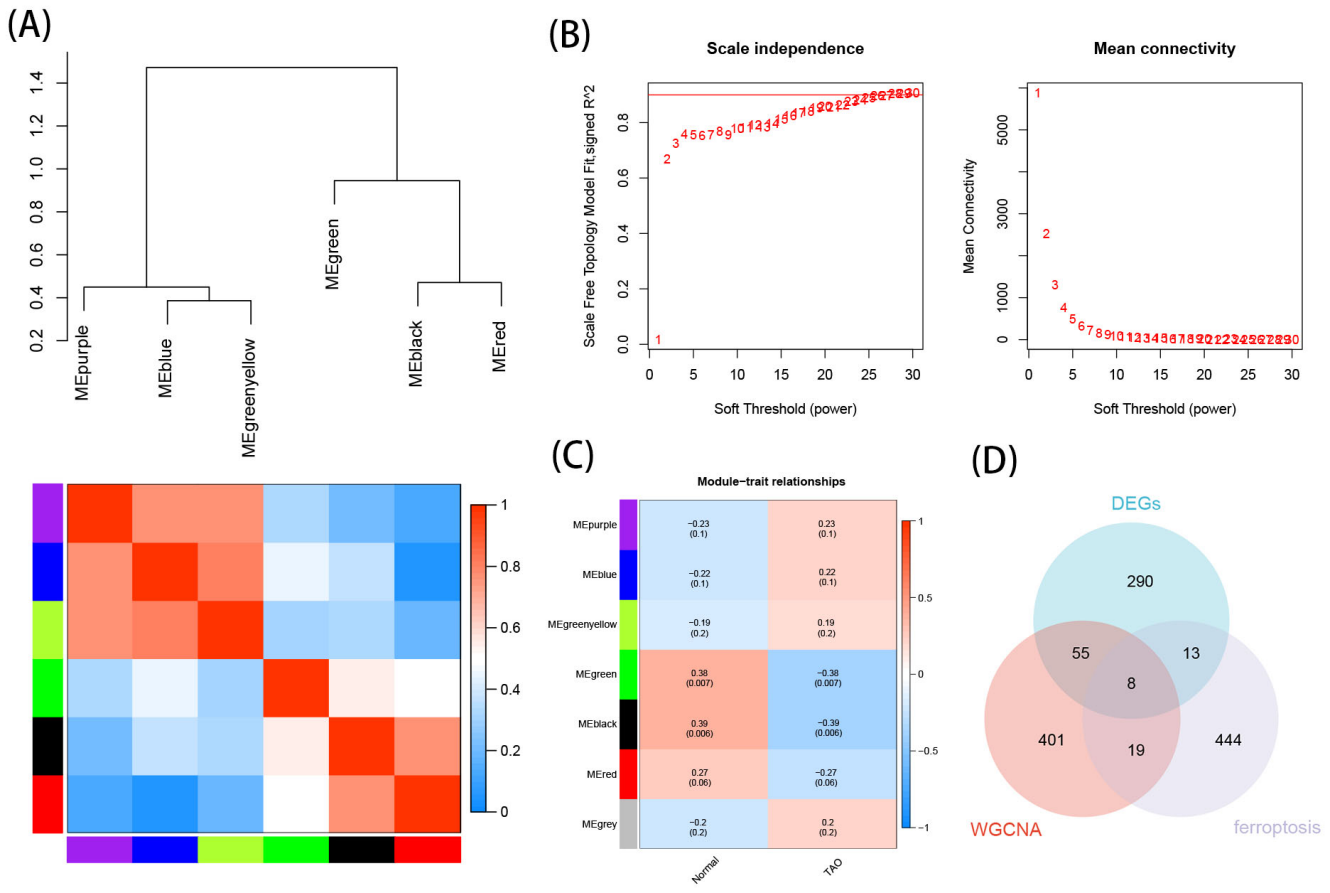
Construction of WGCNA networks and obtain the key genes of ferroptosis in TAO. **(A)** Clustering of module eigengenes, and heat map shows the correlation between each module. **(B)** Scale independence and mean connectivity of various soft-thresholding. **(C)** Module–trait relationships. **(D)** Venn diagram shows the intersection of WGCNA hub genes, EDGs and FRGs.

Based on the soft thresholding method, with β=26, the study found the most consistency in scale-free distribution and gene connectivity (**Figure 4B**). By combining modules with feature factors greater than 0.75, the minimum number of genes in the co-expression network within each module was restricted to 30 (**Figure 4A**). Subsequently, 7 co-expression modules were further detected. MEgreen and MEblack were identified as the two modules with the strongest correlation (**Figure 4C**). From these two modules, 483 genes were selected as hub genes.

### 3.4 TAO key gene for ferroptosis

The intersection of DEGs, hub genes, and ferroptosis-related gene yielded 8 crucial TAO ferroptosis genes, displayed using a Venn diagram (**Figure 4D**).

### 3.5 Acquisition of the OFGs of ferroptosis in TAO

**Figure 5:**
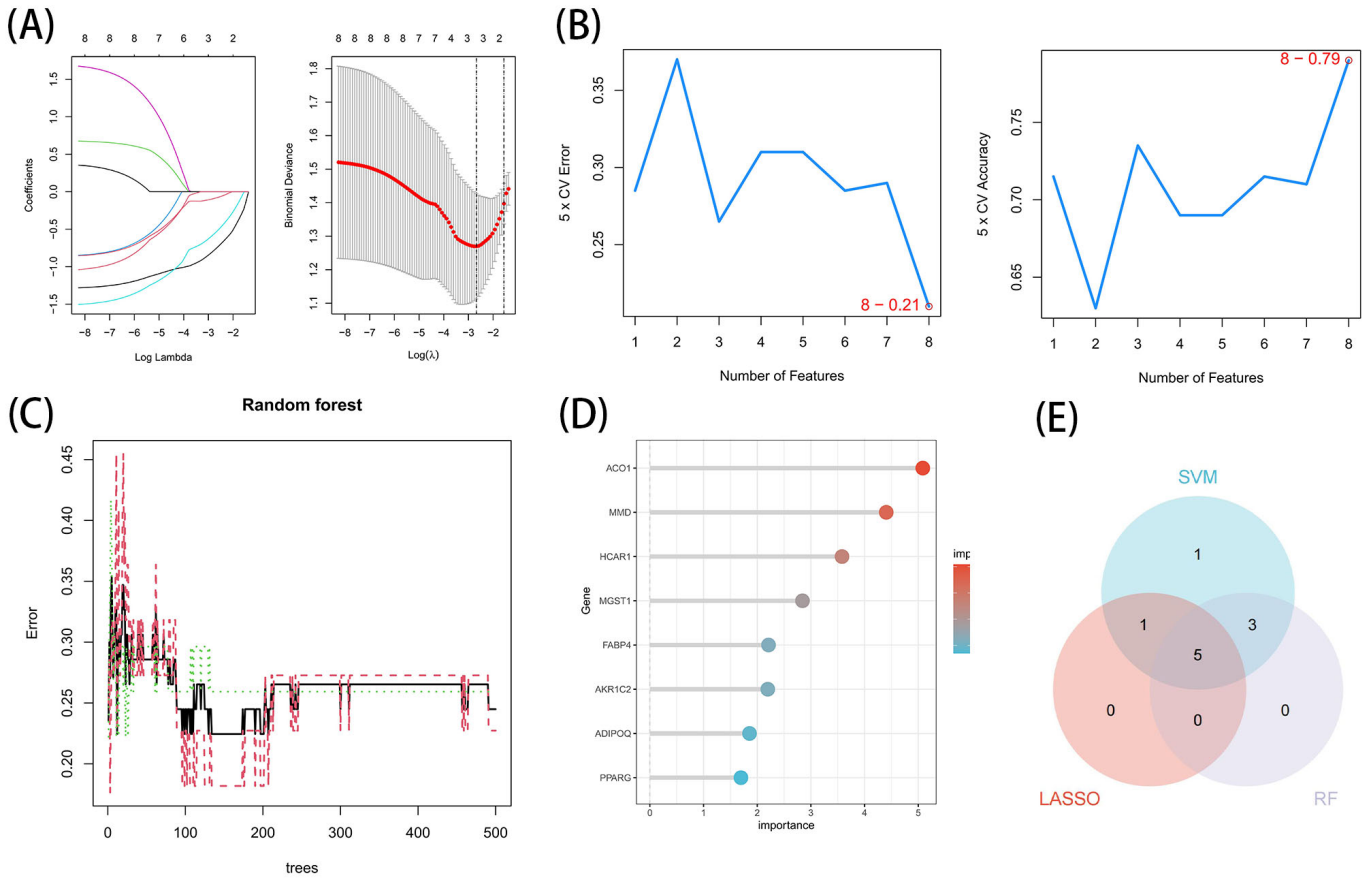
Acquisition of the OFGs of ferroptosis in TAO. **(A)** Biomarker detection using LASSO regression analysis. **(B)** biomarker detection by SVM-REF. **(C, D)** Biomarker detection by random forest. **(E)** Venn diagram shows the shared diagnostic markers of ferroptosis in TAO between LASSO, SVM-REF and random forest.

These 8 key genes were utilized for LASSO, SVM-RFE and RF analysis for further screening. In this study, 3 major biomarkers were identified from the key genes using lassologistio regression method (**Figure 5A**). SVM-RFE method identified 8 genes as important biomarkers (**Figure 5B**). In addition, 6 genes were identified as key biomarkers using RF technology (**Figures 5C, D**). ACO1, HCRA1 and MMD were the overlapping genes of the three methods (**Figure 5E**).

### 3.6 Verification of the OFGs of ferroptosis in TAO

**Figure 6:**
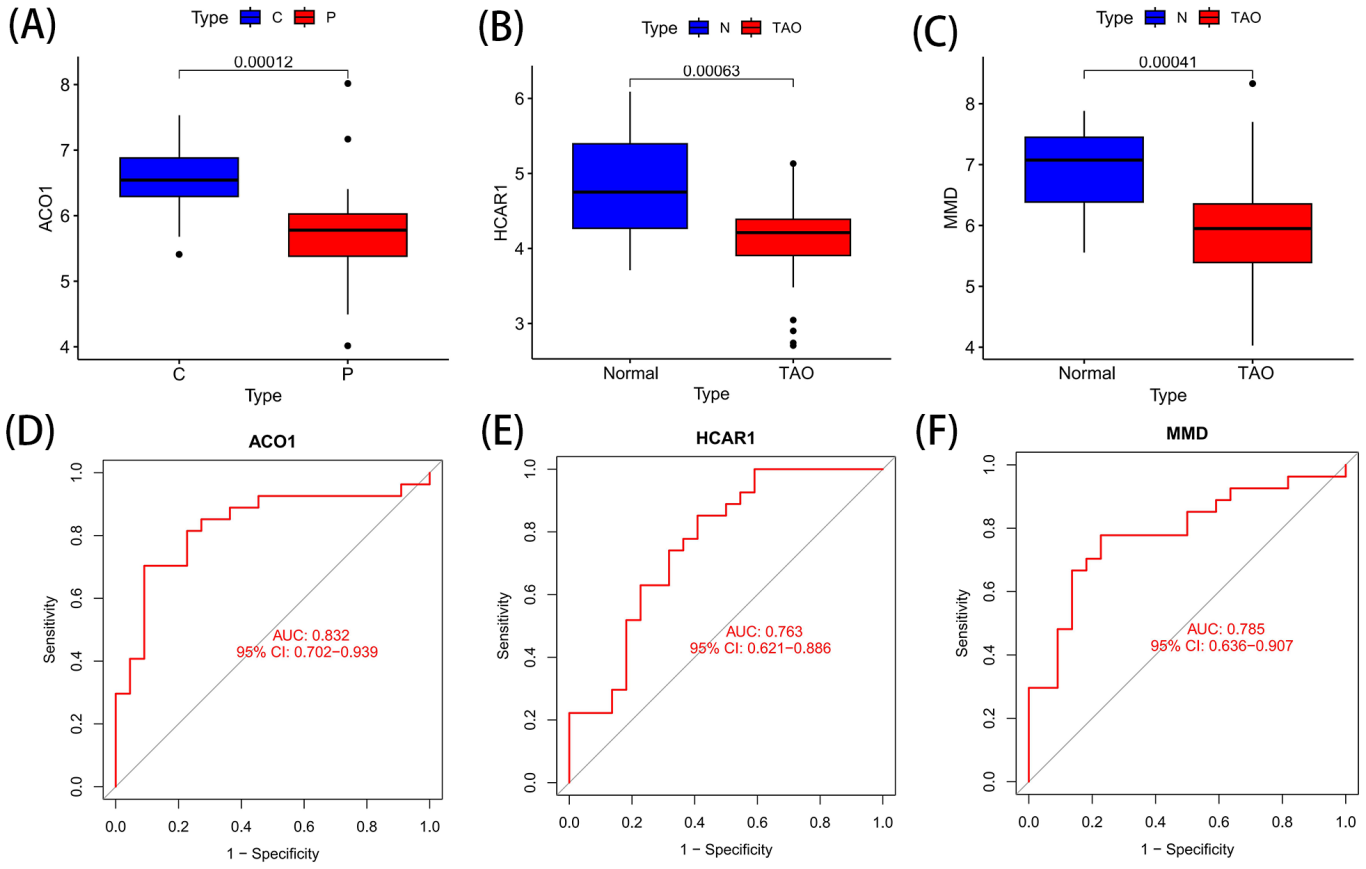
Differential expression and ROC curve of OFGs of ferroptosis. **(A)** The expression difference of ACO1 between TAO and Normal. **(B)** The expression difference of HCRA1 between TAO and Normal. **(C)** The expression difference of MMD between TAO and Normal. **(D)** The predictive value of ACO1 in TAO from the ROC curve. **(E)** The predictive value of HCRA1 in TAO from the ROC curve. **(F)** The predictive value of MMD in TAO from the ROC curve. Each panel displayed the AUC under the curve and 95% CI. ROC, ROC curve; AUC, area under the curve; CI, confidence interval.

ACO1, HCRA1 and MMD are the best characteristic genes of TAO ferroptosis, and these genes are all down-regulated genes (**Figures 6A-C**). The ROC curves of ACO1, HCRA1, and MMD at AUCs of 0.832, 0.763, and 0.785, respectively, showed the possibility of their use as important biomarkers, suggesting that the three bioindicators had high predictive accuracy (**Figures 6D-F**).

### 3.7 GSEA analysis

**Figure 7:**
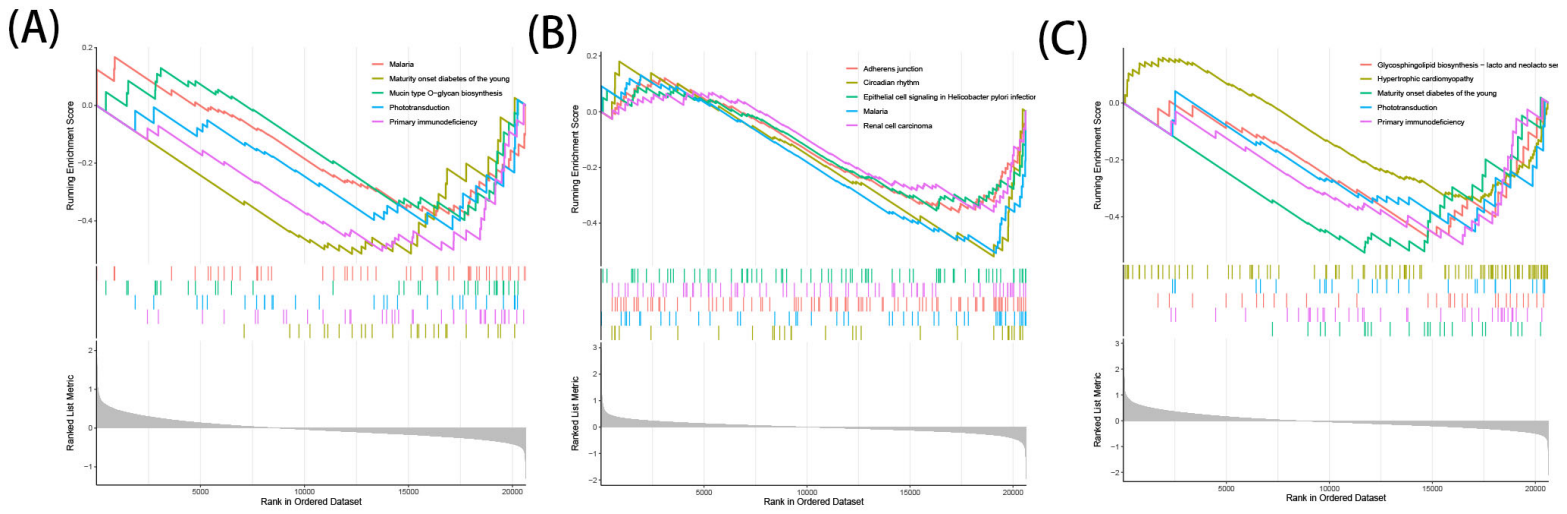
GSEA analysis of OFGs for ferroptosis in TAO. **(A)** GSEA analysis of ACO1. **(B)** GSEA analysis of HCRA1. **(C)** GSEA analysis of MMD.

The downregulation of ACO1 was primarily enriched in pathways related to Primary Immunodeficiency, Phototransduction, Mucin-type O-glycan Biosynthesis, and Malaria (**Figure 7A**). Downregulation of HCAR1 was mainly enriched in Circadian Rhythm, Malaria, Adherens Junction, Renal Cell Carcinoma, and Epithelial Cell Signaling in Helicobacter pylori Infection (**Figure 7B**). The downregulation of MMD was predominantly enriched in pathways associated with Maturity Onset Diabetes of the Young, Primary Immunodeficiency, Glycosphingolipid Biosynthesis - Lacto- and Neolacto-series, Phototransduction, and Hypertrophic Cardiomyopathy (**Figure 7C**).

### 3.8 Immunoinfiltration analysis results

**Figure 8:**
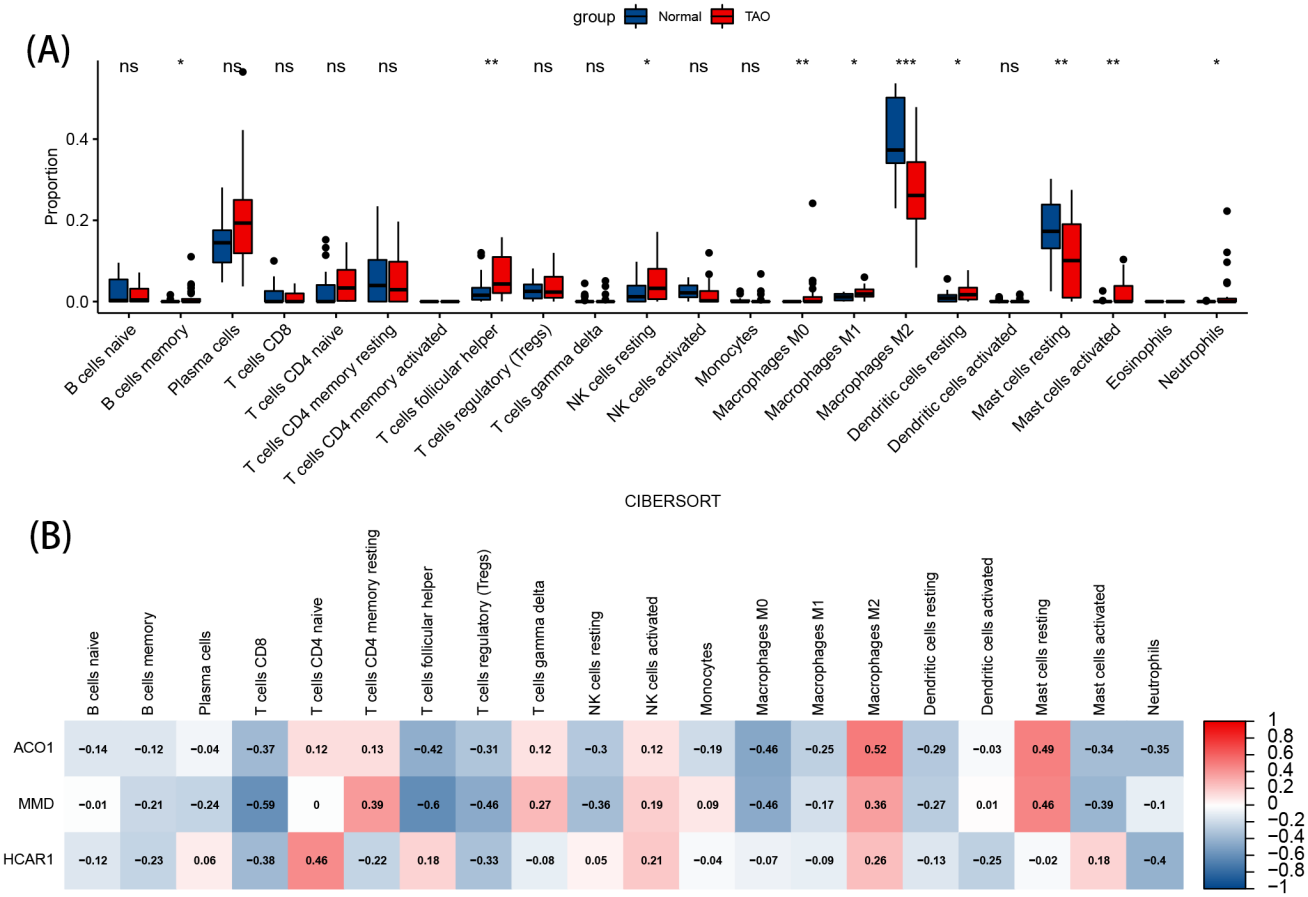
Immune cell infiltration analysis. **(A)** Violin diagram of the proportion of 22 types of immune cells. The red marks represent the difference in infiltration between the TAO and Normal samples. **(B)** Correlation analysis of immune cell infiltrations with three OFGs. Red represents a positive correlation, and blue represents a negative correlation. Darker color implies stronger correlation. *P < 0.05, **P < 0.01, ***P < 0.001. ns, no significance.

The image displays a violin plot of intergroup infiltration differences for 22 types of immune cells, with blue representing normal samples and red representing TAO samples. Among the 22 immune cell types, significant differences in infiltration (P<0.05) were observed in 10 types (**Figure 8A**). Compared to the normal group, the anterior orbital adipose tissue in TAO exhibited increased levels of memory B cells, helper T cells, resting NK cells, M0 macrophages, M1 macrophages, resting dendritic cells, activated mast cells, and neutrophils, while the levels of M2 macrophages and resting mast cells were decreased.

### 3.9 Correlation analysis of OFGs in TAO with infiltrating immune cells

The results of the correlation analysis show that ACO1, HCRA1, and MMD are positively correlated with M2 macrophages. ACO1 and HCRA1 are negatively correlated with helper T cells, resting NK cells, and M0 macrophages, and positively correlated with resting mast cells. Both ACO1 and MMD are negatively correlated with activated mast cells (**Figure 8B**).

### 3.10 Orbital adipose tissue verified FRGS

**Figure 9:**
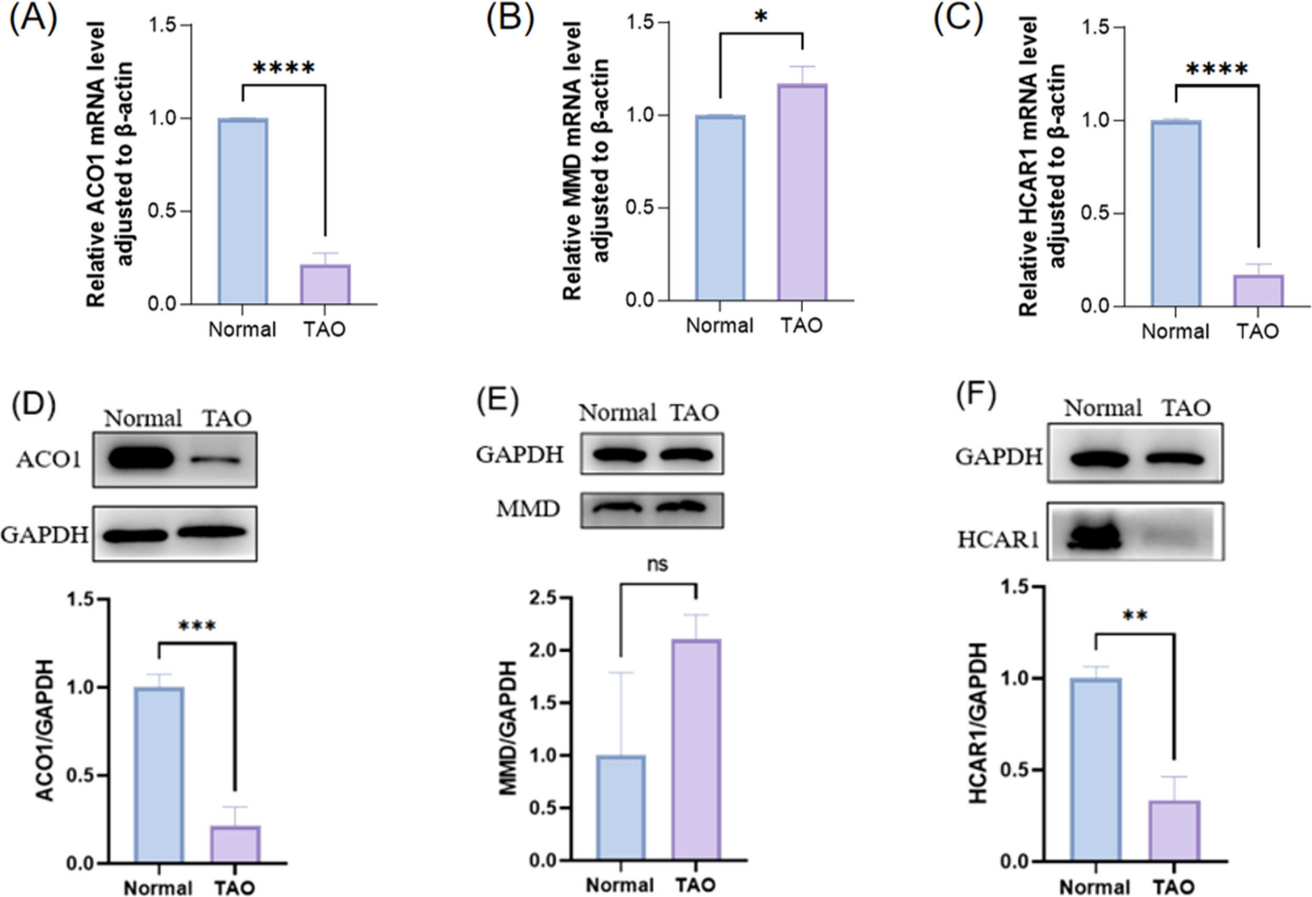
The expression of three FRGS in orbital fat was verified between the healthy control group and the TAO patient group. **(A-C)** The transcription levels of the three FRGS in orbital fat were different. **(D-F)** The protein levels of the three FRGS in orbital fat were different. (Notes: The transcript level expression of these FRGs was normalized to that of β-actin, and the protein level was normalized to that of GAPDH. The statistical significance of differences was calculated by the Student’s t-test. The results are presented as the mean ± SEM. n= 3; ns: p > 0.05,*P < 0.05, **P < 0.01, ***P < 0.001, ****P<0.0001).

To detect the mRNA levels of characteristic genes, the orbital fat discarded during surgery was collected from TAO patients undergoing orbital decompression, and the orbital fat of patients undergoing ocular plastic surgery was used as the control group. Before the follow-up experiment, we performed orbital CT examination on 3 female patients in the TAO group, and the analysis results showed that according to the imaging classification criteria, these 3 patients belonged to the type of muscle hyperplasia as the main manifestation, and all of them were at rest.We verified the expression of differentially expressed FRGs (ACO1, MMD, and HCAR1) in orbital adipose tissue of TAO patients and healthy controls by RT-PCR (**Figures 9A, C**), and the results showed that the expressions of ACO1 and HCAR1 in the TAO group were significantly down-regulated. At the same time, the expression of ACO1 and HCAR1 in the orbital adipose tissue of TAO patients and healthy controls was also significantly low through extraction of orbital adipose tissue of TAO patients (**Figures 9D, F**), which was consistent with the results of our bioinformatics analysis. However, MMD was highly expressed in orbital adipose tissue of TAO patients by both RT-PCR and WB validation results (**Figures 9B, E**), which was contrary to the results of our bioanalysis.

### 3.11 FRGS were verified by cell experiments

**Figure 10:**
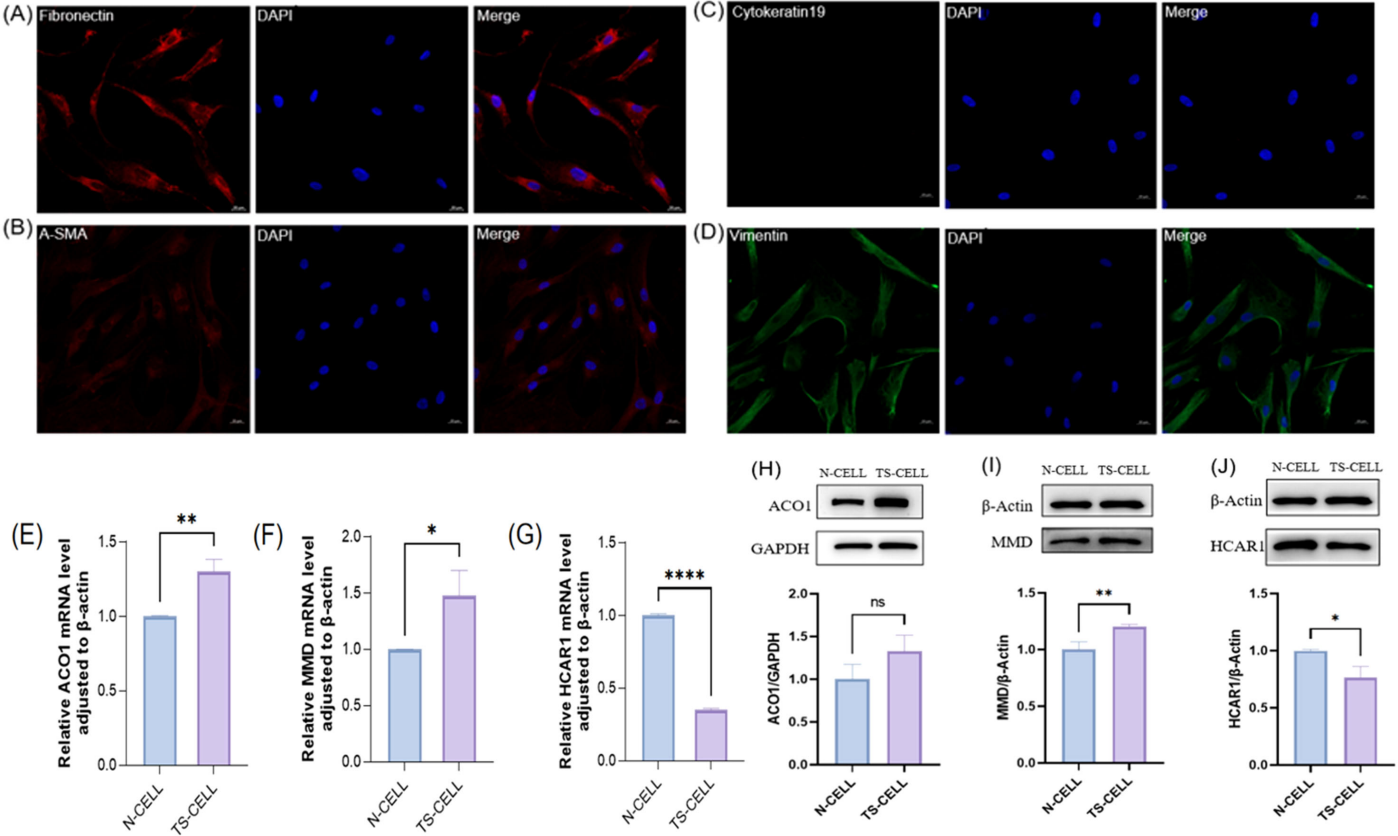
The expression of primary cells from orbital fat of healthy people and TAO patients in the three FRGS was verified. **(A-D)** The primary cells were fibroblasts. **(E-G)** The transcription levels of the three FRGS were different in the primary cells. **(H-J)** The protein levels of the three FRGS were different in the primary cells. (Notes: The transcript level expression of these FRGs was normalized to that of β-actin, and the protein level was normalized to that of GAPDH. The statistical significance of differences was calculated by the Student’s t-test. The results are presented as the mean ± SEM. n= 3; ns: p > 0.05,*P < 0.05, **P < 0.01, ***P < 0.001, ****P<0.0001).

In this study, primary cells were cultured *in vitro* by collecting orbital adipose tissue and identified by cellular immunofluorescence. The results showed that Fibronectin antibody (+), α-SMA antibody (+), Cytokeratin-19 antibody (–), Vimentin antibody (+) (**Figures 10A–D**). The primary cells cultured in this study were orbital adipose-derived fibroblasts (OFs). Further extraction of orbital adipogenic OFs proteins and RNA from normal human orbital adipogenic OFs and TAO patients for FRGS (ACO1, MMD, and HCAR1) verification. In patients with TAO, the expressions of ACO1 and MMD were significantly increased (**Figures 10E, F, H, I**), while the expressions of HCAR1 were significantly decreased (**Figures 10G, J**).

### 3.12 FRGS was verified by cell co-culture *in vitro*

**Figure 11:**
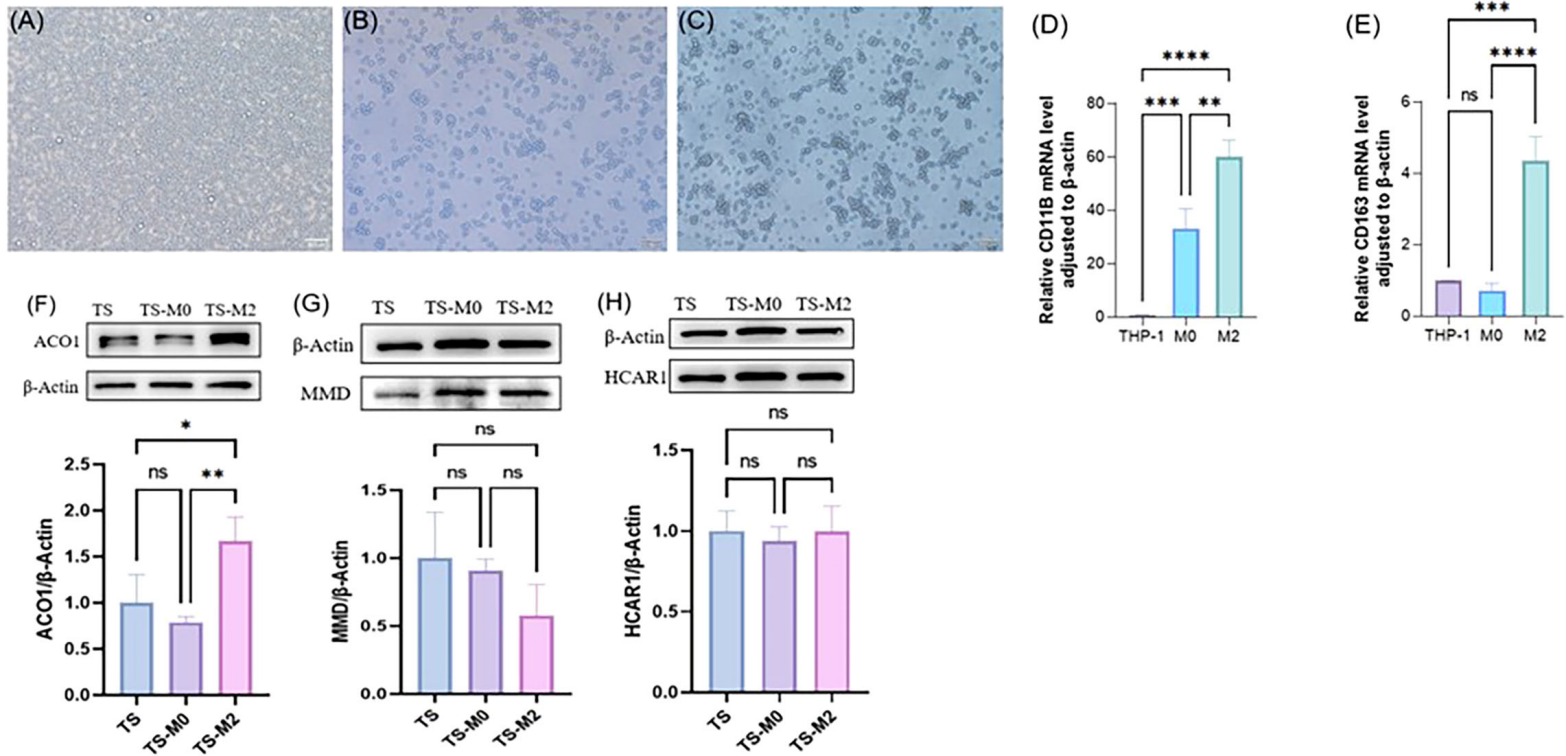
Expression verification of three FRGS after interaction between primary cells from orbital fat of TAO patients and macrophages. **(A)** THP cells. **(B)** M0 macrophages. **(C)** M2 macrophages. **(D)** validation of M0 macrophages. **(E)** validation of M2 macrophages. **(F-H)** Differences in protein levels of three FRGS in primary cell-macrophage interactions after exchange. (The protein levels of these FRGs were normalized to the expression of GAPDH. The statistical significance of differences was calculated by the Student’s t-test. The results are presented as the mean ± SEM. n= 3; ns: p > 0.05,*P < 0.05, **P < 0.01, ***P < 0.001, ****P<0.0001).

THP-1 cells (**Figure 11A**) were induced to differentiate into adherent M0 macrophages by *in vitro* PMA stimulation (**Figure 11B**), and further RT-PCR verification results indicated that CD11b expression was significantly up-regulated after THP-1 induced differentiation (**Figure 11D**). By combining IL-4 with IL-13 to polarize M0, it was found that cell protrusions increased (**Figure 11C**). Further RT-PCR verification results suggested that M0 polarized macrophages appeared as M2 type macrophages, and CD163 expression was significantly up-regulated (**Figure 11E**). Normal DMEM complete medium, M0 supernatant, and M2 supernatant were used to culture orbital adipose OFs, respectively. After 72h, cellular proteins were collected to observe the expression of ACO1, MMD, and HCAR1, indicating that the expression of ACO1 in OFs was significantly increased under the intervention of M2 macrophage conditioned medium (**Figure 11F**). There was no significant difference in the expression of MMD and HCAR1 (**Figures 11G, H**).

## 4 Discussion

TAO is an orbital autoimmune inflammatory disorder commonly associated with thyroid dysfunction. Its pathogenesis is complex, primarily involving the infiltration of inflammatory cells, the production of cytokines, the synthesis of hyaluronic acid, adipogenesis, and myofibrogenesis (6, 7). Ferroptosis is a form of non-apoptotic cell death caused by iron-catalyzed excessive peroxidation of membrane phospholipids, characterized by the presence of several lipid peroxidation enzymes, including acyl-CoA synthetase long-chain family member 4 (ACSL4), lysophosphatidylcholine acyltransferase 3 (LPCAT3), and AGPS, which facilitate the synthesis of polyunsaturated fatty acid (PUFA)-phospholipids (10). Lipid peroxides are produced by lipoxygenases or by ROS generated through the iron-dependent Fenton reaction that extract dienyl propanol hydrogen from PUFA chains of PUFA-phospholipids. Lipid peroxidation is counteracted by several repair systems, notably the xc−/glutathione/glutathione peroxidase 4 (GPX4) system, ferroptosis suppressor protein 1 (FSP1)/CoQ10, and GCH1/BH4 pathways (17, 21–24). However, when one or more of these pathways are inhibited, a free radical chain reaction occurs, leading to ferroptosis.

In this study, we conducted differential expression analysis on the GEO dataset followed by GO and KEGG pathway enrichment analysis of these genes. Based on GO and KEGG enrichment analysis, we preliminarily found that these genes are related to locomotion, localization metabolic process, biological processes such as response to stimulus, and homeostatic process are closely related.Subsequently, we performed WGCNA analysis to identify key modules and intersected genes from these key modules with DEGs to pinpoint critical genes. Additionally, we sourced ferroptosis-related gene datasets from a ferroptosis gene database and intersected these with critical genes to identify ferroptosis-related DEGs in TAO orbital adipose tissue. Using three machine learning algorithms, we selected the best feature genes related to TAO and ferroptosis. The effectiveness of these genes as biomarkers was confirmed by constructing ROC curves and bar graphs, identifying three OFGs (ACO1, MMD, and HCAR1) with AUC values exceeding 0.75, indicating good disease specificity and diagnostic capability. Thus, these DEGs not only suggest the suppression of ferroptosis in TAO tissues and cells but also hold significance for disease biomarkers and potential therapeutic targets, serving as prospective molecular markers for TAO. Expression levels of these three genes were notably lower in the orbital adipose tissue of TAO patients, suggesting that ferroptosis may play a role in orbital adipose tissue remodeling and inflammatory responses in the pathogenesis of TAO. The results of bioinformatics analysis were supplemented by RT-PCR assay of orbital adipose tissue. The results showed that, in addition to MMD, the expression of ACO1 and HCAR1 in orbital connective tissue from patients with TAO decreased significantly at the transcriptional level. At the same time, previous studies have found that OFs cultured in primary orbital connective tissue of TAO patients can inhibit ferroptosis, and these cells have higher proliferation activity after cystine deprivation than those cultured in primary connective tissue of healthy people, and cystine deprivation is one of the pathways inducing ferroptosis (6, 11). This is consistent with the results of this study suggesting that orbital adipose tissue inhibited ferroptosis in TAO patients.In the early stages of TAO pathogenesis, orbital adipose tissue are infiltrated by immune cells, exhibiting various immunoinflammatory changes. Previous immunohistochemical staining results have demonstrated that in the orbital adipose tissue of TAO patients, there is an increased infiltration level of T lymphocytes, B lymphocytes (8, 25), monocytes, macrophages, and mast cells (25). These immune cells release different inflammatory and chemotactic factors, influencing or regulating the proliferation and differentiation of OFs. Our study, utilizing CIBERSORT immune cell infiltration analysis, revealed significant increases in the infiltration levels of memory B cells, follicular helper T cells, NK cells, M0 macrophages, M1 macrophages, resting dendritic cells, activated mast cells, and neutrophils in the orbital adipose tissue of TAO patients, consistent with the findings of previous reports. Importantly, the infiltration of different immune cell subtypes in TAO orbital adipose tissue shows a certain correlation with the expression of ACO1, MMD, and HCAR1 genes in the orbital adipose tissue of TAO patients. Therefore, in this study, we induced M0 and M2 macrophages *in vitro* and co-cultured them with orbital fibroblasts. It was found that the expressions of ACO1, HCAR1, and MMD in M0 macrophages co-cultured with OFs showed a downward trend but no statistical difference, while the expression of ACO1 was significantly increased under the influence of M2 macrophages. The difference was statistically significant. Therefore, we speculated that the infiltration of M2-type macrophages might participate in the occurrence of ferroptosis of orbital OFs.It is also noteworthy that among the 2 OFGs, ACO1 demonstrated the strongest disease diagnostic ability and was the gene with an AUC value greater than 0.8 in the ROC results. Aconitase 1 (ACO1), encoded by ACO1, is an enzyme in the tricarboxylic acid (TCA) cycle and functions as a bifunctional protein with antagonistic roles. When cellular iron levels exceed normal, ACO1 associates with a [4Fe-4S] cluster and acts as an aconitase, catalyzing the conversion of citrate to isocitrate, further producing NADPH to maintain glutathione in its reduced state. Conversely, when cellular iron levels are low, ACO1, upon disassociation from the [4Fe-4S] cluster, acts as Iron Regulatory Protein 1 (IRP1) (26–28). ACO1 and IRP1 are involved in the regulation of iron homeostasis in cells or systems, thus regulating the occurrence of ferroptosis (Annex 1). Currently, dysregulation of iron homeostasis has been found to be an important pathogenesis of some tumors, neurodegenerative diseases, metabolic diseases, and autoimmune diseases (29, 30).Tang et al. have demonstrated that knockdown or inhibition of ACO1 expression significantly weakens the anti-tumor and ferroptosis-inducing effects of curcumin (31). In our study, a significant downregulation of ACO1 in the orbital adipose tissue of TAO patients was observed, suggesting an inhibition of ferroptosis in TAO orbital adipose tissue and cells. Given the function of the ACO1 gene, its reduced expression indicates a decrease in cellular ACO1 and IRP1 proteins, leading to insensitivity to intracellular iron levels, consistent with previous studies that have shown a higher ferroptosis tolerance in TAO patients’ OFs. The expression of ACO1 may also be influenced by the immune microenvironment in TAO orbital adipose tissue. Correlation analysis with immune cells revealed that ACO1 has significant correlations with resting mast cells, M2 macrophages, M0 macrophages, and follicular helper T cells. Meanwhile, MO and M2 macrophages constructed *in vitro* were co-cultured with orbital OFs, and it was found that M2 macrophages may be involved in regulating the expression of ACO1 in orbital OFs, thus promoting the occurrence of ferroptosis.MMD (Monocyte to Macrophage Differentiation Associated), also known as Adipo-Q Receptor 11 (PAQR11), is one of the lesser-studied members of the Progestin and PAQR family (32). Functioning as a complete membrane scaffolding protein, it facilitates vesicular transport, mitotic signal transduction, and metastatic growth (33, 34). Phadnis et al. showed that MMD interacts with ACSL4 and MBOAT7 to promote the entry of arachidonic acid into phosphatidyl inositol (PI), increasing cell sensitivity to lipid metabolism and **ferroptosis** (Annex 1) (35). Consequently, reduced expression of MMD diminishes cellular sensitivity to lipid metabolism and ferroptosis, inhibiting ferroptosis. Correlation analysis between MMD expression and immune cells indicates that, aside from resting mast cells, MMD is significantly negatively correlated with CD8+ T cells, T regulatory cells (Tregs), M0 macrophages, and follicular helper T cells. In this study, it was found that the expression of MMD was up-regulated in both orbital adipose tissue and orbital adipogenic OFs of TAO patients, which may also be related to the small number of biological samples of corticosteroid treatment and analysis

This study also found that the expression of HCAR1, a gene associated with ferroptosis, was down-regulated in both orbital adipose tissue and orbital adipose OFs of TAO patients. HCARs are part of a family of GPCRs (G protein-coupled receptors) activated by intermediates of energy-producing metabolic pathways (36). HCAR1 is a regulatory protein involved in various physiological processes, including the modulation of metabolic pathways, and is linked to the pathogenesis of numerous diseases such as neurological disorders, vascular diseases, inflammatory conditions, ocular diseases, cardiovascular diseases, myeloproliferative disorders, and cancers (37–40). Previous studies have identified HCAR1 as an expression regulator of monocarborxylat transporter 1(MCT1), activated by lactate binding, which triggers downstream signaling pathways leading to the upregulation of MCT1 expression, thereby enhancing lactate uptake in cells (41, 42). Huang et al. have indicated that HCAR1, as a lactate receptor, can activate the downstream PI3K/AKT pathway, subsequently upregulating NADPH oxidase 4 (NOX4) to promote the production of ROS and chondrocyte damage (43). Differential expression analysis of genes associated with TAO and ferroptosis showed that low expression of HCAR1 in TAO patients may inhibit orbital adipose tissue and cell ROS production, thereby inhibiting cell ferroptosis (Annex 1). However,HCAR1 is highly expressed in human adipose tissue, and its transcription is largely regulated by metabolic factors, including peroxisome proliferator-activated receptor gamma (PPAR-γ), which is one of the proximal promoters of HCAR1 and can induce the receptor through PPAR-γ transcriptional activation (44, 45). Clinically, the orbital histological changes of TAO patients showed inflammation and remodeling of orbital connective tissue, and TAO patients showed both orbital muscle fibroplasia and orbital fat hyperplasia, which may lead to inconsistent expression of HCAR1 in patients with different types of TAO. The patients selected in this verification process were all TAO patients with quiescent muscle fibrosis, whose expression was significantly down-regulated.

This study has several limitations. Firstly, the high-throughput chips and sequencing data utilized were sourced from public databases, limiting the availability of comprehensive patient-related information such as age, gender, duration of TAO, prior treatment history, and clinical activity scores. This constraint impedes the establishment of robust associations between gene expression patterns and clinical phenotypes of the disease. In addition, the patient tissue samples selected in this gene verification were limited, and the gene expression could not directly correspond to the corresponding disease phenotype, and the orbital adipose tissue of clinical TAO patients was not collected for immunohistochemistry, which can not directly reflect the influence of different subtypes of immune cells on the expression of FRGS. Next, we will further explore the regulatory mechanisms of these genes in ferroptosis and the biological functions of TAO pathogenesis.

This study identified two OFGs of ferroptosis (ACO1 and HCAR1) in patients with TAO, which holds significant implications for future research on the role of ferroptosis in TAO. Furthermore, ROC curve analysis and RT-qPCR demonstrated that these genes possess disease-specific and robust diagnostic capabilities, suggesting their potential as molecular biomarkers for the disease. Additionally, CIBERSORT analysis revealed a close association between these genes and infiltrating immune cells in TAO orbital adipose tissue, indicating their potential as therapeutic targets warranting further investigation.

## Data Availability

The original contributions presented in the study are included in the article/supplementary material. Further inquiries can be directed to the corresponding authors.
